# Coordinated Dialogue between UHRF1 and DNMT1 to Ensure Faithful Inheritance of Methylated DNA Patterns

**DOI:** 10.3390/genes10010065

**Published:** 2019-01-18

**Authors:** Christian Bronner, Mahmoud Alhosin, Ali Hamiche, Marc Mousli

**Affiliations:** 1 Institut de Génétique et de Biologie Moléculaire et Cellulaire (IGBMC), Université de Strasbourg, INSERM U1258 CNRS UMR 7104, 1 rue Laurent Fries, 67404, Illkirch, France; hamiche@igbmc.fr; 2 Department of Biochemistry, Faculty of Science and Cancer and Mutagenesis Unit, King Fahd Medical Research Center, King Abdulaziz University, Jeddah, Saudi Arabia; malhaseen@kau.edu.sa; 3 Laboratoire de Bioimagerie et Pathologies, Faculté de Pharmacie, Université de Strasbourg, CNRS UMR 7021, 67401 Illkirch, France; marc.mousli@unistra.fr

**Keywords:** cell identity, DNA methylation, DNMT1, epigenetics, gene expression, UHRF1

## Abstract

DNA methylation, catalyzed by DNA methyltransferases (DNMTs), is an epigenetic mark that needs to be faithfully replicated during mitosis in order to maintain cell phenotype during successive cell divisions. This epigenetic mark is located on the 5′-carbon of the cytosine mainly within cytosine–phosphate–guanine (CpG) dinucleotides. DNA methylation is asymmetrically positioned on both DNA strands, temporarily generating a hemi-methylated state after DNA replication. Hemi-methylation is a particular status of DNA that is recognized by ubiquitin-like containing plant homeodomain (PHD) and really interesting new gene (RING) finger domains 1 (UHRF1) through its SET- (**S**u(var)3-9, **E**nhancer-of-zeste and **T**rithorax) and RING-associated (SRA) domain. This interaction is considered to be involved in the recruitment of DNMT1 to chromatin in order to methylate the adequate cytosine on the newly synthetized DNA strand. The UHRF1/DNMT1 tandem plays a pivotal role in the inheritance of DNA methylation patterns, but the fine-tuning mechanism remains a mystery. Indeed, because DNMT1 experiences difficulties in finding the cytosine to be methylated, it requires the help of a guide, i.e., of UHRF1, which exhibits higher affinity for hemi-methylated DNA vs. non-methylated DNA. Two models of the UHRF1/DNMT1 dialogue were suggested to explain how DNMT1 is recruited to chromatin: (i) an indirect communication via histone H3 ubiquitination, and (ii) a direct interaction of UHRF1 with DNMT1. In the present review, these two models are discussed, and we try to show that they are compatible with each other.

## 1. Introduction

### 1.1. DNA Methylation Patterns: Layers of Epigenomes

DNA methylation mainly occurs on cytosine–phosphate–guanine (CpG) dinucleotides, and almost 70–80% of all CpG dinucleotides (~3 × 10^7^) are methylated in human cell genomes [1,2]. DNA methylation can also occur at non-CpG, including cytosine–phosphate–adenine (CpA), cytosine–phosphate–thymine (CpT), and cytosine–phosphate–cytosine (CpC), with the first one being the most frequent [3]. The role of DNA methylation is still a matter of debate [3]. Considering that de novo DNA methyltransferases (DNMTs), such as DNMT3A and DNMT3B, catalyze methylation at non-CpG sites, we do not extend this review to non-CpG methylation. Although the role of DNA methylation was reviewed elsewhere, before going deeper into the insights of the mechanism of DNA methylation pattern duplication, a few reminders on the location of the methylated CpG sites are made hereafter.

Methylated CpG dinucleotides are preferentially localized within repetitive elements (SINEs; Short Interspersed Nuclear Elements, LINEs; Long INterspersed Nuclear Elements, and LTR; Long Terminal Repeat), centromeres, and coding regions of functional genes (gene bodies) [4]. Methylation of CpG dinucleotides in these areas guarantees genomic stability and prevents aberrant initiation of transcription [5,6]. The primary well-recognized role of DNA methylation lies in transcriptional repression of retrotransposons, X chromosome inactivation, and mono-allelic expression of imprinted genes [7]. Therefore, the methylation of DNA, along with enhancers and gene bodies [8,9,10], is involved in gene expression regulation. Non-methylated CpG dinucleotides are frequently observed in promoter regions of active genes where they form “CpG islands (CGIs)”. CpG islands are regions greater than 550 bp of DNA with a GC content greater than 50%, and an observed CpG/expected CpG ratio greater than 0.6 [11]. Approximately, 70% of human gene promoters contain CGIs, usually unmethylated in normal cells, thus allowing transcription of the corresponding gene. During development and differentiation, around 6% of them undergo methylation in a tissue-specific manner, thereby contributing to the occurrence of different phenotypes [5,11]. Irregularities in methylation pattern, especially in promoters, can lead to profound abnormalities including malignant transformation of cells [4]. In cancers, two opposite patterns of DNA methylation are observed and contribute together to tumorigenesis. Firstly, CpG islands in promoter regions of various tumor suppressor genes (TSGs), such as *p16^INK4A^*, *p73*, *BRCA1*, and *TIMP3*, are hypermethylated in cancer, inhibiting their expression and, thus, leading to uncontrolled proliferation of cancer cells [12,13,14,15]. Nonetheless, establishment of this methylation pattern is not yet deciphered. Secondly, a global hypomethylation is observed in cancer cells, which promotes the expression of oncogenes and aggravates tumorigenesis by inducing genomic instability [16,17]. The origin of these two upheavals in DNA methylation patterns is not yet fully elucidated, but might find a beginning of an explanation in the altered communication between ubiquitin-like containing plant homeodomain (PHD) and really interesting new gene (*RING*) finger domains 1 (UHRF1) and DNA methyltransferase 1 (DNMT1) [18,19], which is the matter of the present review. However, what is clear is that these DNA methylation patterns are faithfully transmitted from a mother cancer cell to the two daughter cancer cells with a phenotype preserved almost to infinity. Considering that cancer cells usually divide faster than normal cells and that they have a more stable phenotype throughout successive mitosis, especially under in vitro culture conditions [20], one might imagine that the DNMT1/UHRF1 tandem is more efficient in cancer cells than non-cancerous cells.

Efforts over the past decade shed light onto the dynamics and the molecular mechanisms underlying DNA methylation pattern duplication occurring after DNA replication. One important breakthrough was achieved by the discovery of the UHRF1/DNMT1 network.

### 1.2. The Role of UHRF1/DNMT1 Tandem

Chromosomal mapping of human *UHRF1* and *DNMT1* genes both at 19p13, more specifically at 19p13.3 and 19p13.2, respectively, [21] and genome sequencing revealed that they are separated by about 50 Mb, i.e., 50 centimorgans (personal observations). It is worth noting that the tissue-specific expression of UHRF1 and DNMT1 is tightly linked, suggesting that they need each other to exert their role. Indeed, for both genes, the most elevated expressions were found in the appendix, bone marrow, lymph node, and testis [22,23]. In contrast, highly differentiated tissues, such as the heart, liver, pancreas, prostate, and salivary glands, were among the tissues that express the lowest levels of *DNMT1* and *UHRF1* messenger RNAs (mRNAs) [22,23]. This may explain that *UHRF1^−/−^* phenocopies *DNMT1^−/−^* [24,25], that *DNMT*^−/−^ is embryonic lethal at day 9 [26], and that UHRF1^−/−^ is mid-gestational lethal [27], suggesting that both genes have interdependent roles in DNA methylation maintenance. This also suggests that both genes might have similar pathways of regulation. Pharmacological or pathological regulation of the expression of *UHRF1* and *DNMT1* genes was extensively reviewed elsewhere [22,28,29,30,31,32,33,34,35,36,37]. Briefly, one interesting point with deep impact is that downregulation of *UHRF1* and/or *DNMT1* always allows re-expression or enhanced expression of a large number of tumor suppressor genes, including *RB1*, *p16^INK4A^*, *CDH13*, *SHP1*, *SOCS3*, *3OST2*, *BRCA1*, *CDX2, RUNX3, FOXO4, PPARG, PML*, *MEG3, HHIP*, *IGFBP3*, *SFRP1*, and *14-3-3σ* [29,34,35,38,39,40,41,42,43,44,45]. Also of note, it was shown that UHRF1 is involved in epigenetic silencing of *KiSS1,* a metastasis suppressor gene [46]. The mechanism of the re-expression of tumor suppressor genes following decreased expression of *DNMT1* and *UHRF1* remains elusive. Indeed, it is clear that these two proteins are involved in the maintenance of hypermethylation of promoters, but how they are demethylated remains a mystery. A passive demethylation, via a downregulation of *UHRF1*, through successive mitosis appears unlikely, as *UHRF1* is indispensable for cell proliferation [22,47].

For global DNA hypomethylation, a diminished interaction was suggested between *UHRF1* and *DNMT1* [18,19], although *UHRF1* expression is enhanced in all cancers so far investigated [29,34,35,36,48]. However, a ubiquitin-dependent degradation of *DNMT3A* induced by *UHRF1* and/or *UHRF2* might also be involved [49]. Indeed, considering that DNMT3A is involved in de novo DNA methylation, an increase of UHRF1 through the targeting of DNMT3A also likely contributes to the global DNA hypomethylation in cancer cells [49].

The maintenance of DNA methylation at the replication fork is believed to be ensured by the DNMT1/PCNA (Proliferating Cell Nuclear Antigen) tandem [50,51,52,53]; however, surprisingly, its disruption exerts little effect on genomic DNA methylation in contrast to that of the UHRF1/DNMT1 tandem, which induces massive DNA hypomethylation [18]. Furthermore, the occurrence of a disruption in the oncogenic process was extended to the UHRF1/DNMT1/PCNA complex [19]. Complementary to this event, it was demonstrated that UHRF1 overexpression drives DNA hypomethylation by delocalizing DNMT1 [54], which further supports that abnormal cooperation within the UHRF1/DNMT1 tandem may be one of the first steps of tumorigenesis onset. Indeed, the de novo methylation of genes frequently observed in cancers could be catalyzed by DNMT1, rather than by DNMT3A or DNMT3B [26,55,56].

At the molecular level, the role of UHRF1 can be summarized in the targeting of DNMT1 to replication forks by serving as a guide for DNMT1 at hemi-methylated CpG sites [25,57,58,59,60,61,62,63,64]. At the cellular level, the UHRF1/DNMT1 tandem is involved in many processes, including differentiation [65], cell senescence [66], stem cell self-renewal [67,68,69], neurogenesis [70], germinal center B-cell expansion [71], maturation of colonic T lymphocytes [72], smooth muscle plasticity [73], and induced pluripotent stem-cell reprogramming [74] and development [75,76].

### 1.3. UHRF1 and DNMT1, Interdependent Multi-Domain Proteins

**Figure 1:**
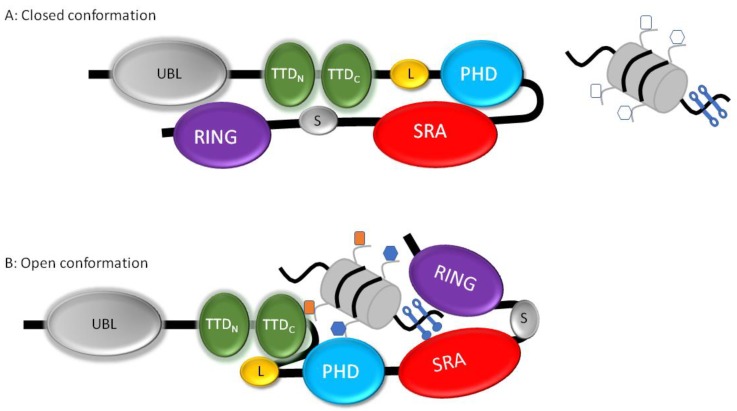
Schematic representation of ubiquitin-like containing plant homeodomain (PHD) and really interesting new gene (RING) finger domains 1 (UHRF1)’s domains with open and closed conformations. (**a**) Closed conformation of UHRF1. In the absence of methylated DNA (open lollipops), UHRF1 adopts a closed conformation with the spacer (S) facing the tandem Tudor domain (TTD) and the RING domain facing the ubiquitin-like domain (UBL). This interaction may represent a kind of auto-inhibitory mechanism as UBL exhibits analogy with ubiquitin. The SET- and RING-associated (SRA) domain faces the PHD domain. The linker (L) is the sequence between the TTD and PHD. (**b**) Open conformation of UHRF1. In the presence of hemi-methylated DNA (full lollipops on one strand) and in the presence of di/trimethylated lysine 9 on histone H3 (ocher rectangle on nucleosome) and of unmodified arginine 2 on histone H3 (blue hexagon on nucleosome), UHRF1 adopts an open conformation allowing each domain to fulfill its respective role. For instance, the RING domain can ubiquitinate histone H3 and the TTD with methylated histone H3.

UHRF1 is a multi-domain protein (Figure 1) including a ubiquitin-like domain (UBL), a tandem Tudor domain (TTD), a plant homeodomain (PHD), an SET- and RING-associated (SRA) domain, and a really interesting new gene (RING) domain, with the latter domain conferring the sole enzymatic activity [77]. Two-thirds of the primary sequence of UHRF1 contributes to these structural domains, suggesting that this protein fulfils multiple important roles. One of these roles is the link between the histone code and DNA methylation. Indeed, SRA is able to sense the presence of hemi-methylated DNA through its SRA domain and to recognize via the tandem Tudor domain, probably at the same time, two or three methyl groups on lysine 9 of histone H3 (H3K9me2/3) [25,61,64,78,79,80,81,82]. Accordingly, it was shown that UHRF1 can target DNMT1 for DNA methylation maintenance via binding to H3K9me2/3 or hemi-methylated CpG [83], thus ensuring a kind of security for the faithful inheritance of DNA methylation and, probably reciprocally, histone H3 methylation profiles. The PHD domain is involved in binding to unmodified H3R2 [84,85,86,87] and, thus, contributes to the binding of UHRF1 to chromatin [88]. The role of the UBL might allow UHRF1 to follow the proteasome pathway [89] and/or be involved in the interaction with the E2 conjugate to promote E3 ligase activity [90,91].

DNMT1 is a large protein of 1616 amino acids (aa) [31,92,93,94,95] composed of a large regulatory N-terminal region (1000 aa) and a catalytic C-terminal region including 10 catalytic motifs that are important for the interaction with *S*-adenosyl methionine, the methyl group donor. The C-terminal and N-terminal regions are linked by six Lys/Gly (KG) dipeptide repeats. The N-terminal region is composed of (i) a domain able to interact with a large panel of proteins, (ii) a replication focus targeting sequence (RFTS) domain, which is a matter of very active research over the last few years, involved in the recruitment of DNMT1 to the DNA replication fork, (iii) a zinc-binding domain, (iv) two bromo-adjacent homology (BAH) domains, and (v) a nuclear localization signal (aa 191–211). The crystal structure revealed that the RFTS domain directly associates with the catalytic domain, thereby exhibiting auto-inhibition ability [96].

## 2. The UHRF1/DNMT1 Dialogue on Chromatin

### 2.1. The Ubiquitination of Histone H3 by UHRF1 as a Chromatin Anchorage for DNMT1: Model A

It was previously shown by several groups [96,97,98,99] that UHRF1 adopts a closed conformation with a C-terminal part (spacer aa 586–674) facing the TTD, and the SRA domain facing the PHD domain (Figure 1a). This spacer region contains a polybasic region (PBR) in the C-terminus, which prevents the recognition of H3K9me3 by the TTD domain. Therefore, in this conformation, it appears that UHRF1 is unable to bind to H3K9me2/3 via its TTD, to the unmodified R2 of histone H3 via the PHD domain, or to hemi-methylated DNA via its SRA domain [97]. However, interestingly, the presence of hemi-methylated DNA impairs the intramolecular interactions, and each domain can exert its ability to interact with its selective target (Figure 1b). It was further suggested that the docking of TTD to H3K9me3 may induce the ubiquitination activity of the RING domain toward lysines 14, 18, and 23 (K14, K18, K23) of histone H3 [97], thus giving rise to a signal for DNMT1 recruitment [100,101,102,103]. Fine-tuned regulation of H3 ubiquitination suggests that the RING finger of UHRF1 induces mono-ubiquitination of histone H3 at K14, K18, and K23 but not polyubiquitination [88,102] in spite of its ability to catalyze it [104,105]. Apparently, two mono-ubiquitinated lysines are enough for DNMT1 to bind histone H3, i.e., H3K14/K18, H3K14/K18, or H3K18/K23, with the help of the N-terminal tail of histone H3 to confer high affinity [102]. It is the RFTS domain of DNMT1 that is involved in this anchorage with similar affinities for each double mono-ubiquitinated motif [102]. The mode of ubiquitin binding is, however, different from other reported recognition patterns [106,107]. Regarding the high affinity of DNMT1 for ubiquitinated histone H3, it was suggested that this interaction gives robustness to the inheritance of DNA methylation patterns [106]. It was further proposed that these lysines could be crucial for gene expression regulation as they can be ubiquitinated (repressive effect) or acetylated by p300/CBP or GCN5 (General Control Of Amino Acid Synthesis Protein 5) which are enzymes involved in gene transcription [108].

In accordance with a role of histone H3 ubiquination in DNA methylation maintenance, depletion of ubiquitin-specific peptidase 7 (USP7) in HeLa cells enhanced histone H3 ubiquitination and led to enhancement of DNMT1 nuclear foci during DNA replication [109]. However, it was shown that USP7 stimulates both the maintenance and de novo DNA methylation activity of DNMT1 in vitro [88,110], further supporting a complex role of USP7 in DNA methylation maintenance that remains to be fully deciphered.

### 2.2. Domain–Domain Interactions between DNMT1 and UHRF1: Model B

**Figure 2:**
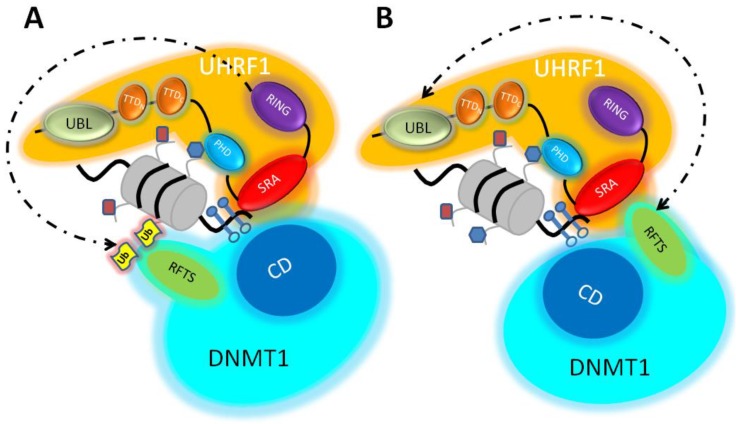
Dialogue model between UHRF1 and DNA methyltransferase 1 (DNMT1) in the presence of hemi-methylated DNA. (**a**) Model A: targeting of DNMT1 to chromatin via histone H3-dependent ubiquitination. Two molecules of ubiquitin (Ub) on the nucleosome, mediated by the RING domain of UHRF1 (dotted line), serve as an anchorage for DNMT1 via the replication focus targeting sequence (RFTS) domain. This interaction allows the alleviation of the auto-inhibitory activity of RFTS on the catalytic domain (CD) of DNMT1. Concomitantly, the TTD binds di/trimethylated lysine 9 on histone H3 (ocher rectangle on nucleosome) and the PDH binds unmodified arginine 2 on histone H3 (blue hexagon on nucleosome). (**b**) Model B: targeting of DNMT1 to chromatin via UHRF1 domain interactions. The UBL (dotted line) and SRA domain interact with the RFTS domain of UHRF1, allowing the release of the catalytic domain (CD) of DNMT1. As with model A, the TTD binds di/trimethylated lysine 9 on histone H3 (ocher rectangle on nucleosome) and the PDH binds unmodified arginine 2 on histone H3 (blue hexagon on nucleosome).

There were numerous reports [24,25,60,111,112,113] showing a physical interaction between UHRF1 and DNMT1, leaving little doubt that UHRF1 communicates directly with DNMT1. Initially, two independent groups reported the interaction between UHRF1 and DNMT1 [24,60]. Domain mapping nevertheless showed some discrepancies. Indeed, we observed that the human SRA domain of UHRF1 is responsible for the interaction with human DNMT1 [60] (Figure 2b). The other group found that it is rather the PHD domain of mouse UHRF1 that is involved in DNMT1 interaction [24]. Additionally, it was recently reported that the UBL domain of UHRF1 is able to bind to the RFTS domain of DNMT1 (N-terminal region), as well with the amino-acid sequence encompassing aa 621–1616 [91,106]. The latter interaction is involved in the stimulation of the enzymatic activity of DNMT1, whereas the first (RFTS) is rather implicated in the alleviation of the auto-inhibition exerted by DNMT1 [91,106]. Three regions of DNMT1 were shown to bind to UHRF1, namely amino-acid residues 1–446, 1081–1408 in mouse [24], and 401–615 in human [60]. We previously suggested that the discrepancies might come from the species-specific difference [59]; however, it was later shown that the interacting domain in mouse DNMT1 was a region encompassing aa 291–601 corresponding to the RFTS domain [114]. In accordance, it was shown that the interacting motif of DNMT1 with UHRF1 is a region spanning from aa 380–399 [88]. Interestingly and consistently, the spacer of UHRF1, located beside the SRA domain, finds an important role as it facilitates the interaction of the SRA domain with hemi-methylated DNA and the interaction with DNMT1 [97]. It should be mentioned here that the spacer might explain the different functions of UHRF1 and UHRF2, as the latter lacks this sequence, as well as DNA methylation maintenance capacity [111,115,116]. Therefore, the most likely sites of interaction are the SRA domain and the UBL of UHRF1, with the RFTS domain of DNMT1 (Figure 2). This multiple site connection might establish a kind of dialogue between these two proteins depending on the methylation status of the DNA and/or the motion of the complex along the DNA. In accordance with this, it was shown that full-length UHRF1, the SRA domain [117], or the UBL [106] increase the preference or catalytic activity of DNMT1 for hemi-methylated DNA. This effect is dependent on the RFTS domain of DNMT1. Indeed, the RFTS of DNMT1 exerts inhibitory action on its catalytic domain, by occupying the DNA binding pocket, as shown by crystallography [112,113]. This could be evidenced by incubating DNMT1 with UHRF1 or the SRA domain prior to the addition of DNA, as UHRF1 competes with DNMT1 for binding to DNA. When DNA is added first, UHRF1 binds to DNA and does not reverse the clamped conformation of DNMT1. A model in which UHRF1 binds to hemi-methylated CpG site via its SRA domain with subsequent recruitment of DNMT1 was suggested [117]. This model supposes that UHRF1, through the SRA domain, binds firstly to DNA, which is in accordance with other studies proposing that the SRA domain moves along DNA to seek for the presence of hemi-methylated DNA [57,58]. In accordance with a histone-independent recruitment of DNMT1, it was intriguingly proposed that auto-ubiquitination of UHRF1 might serve as a docking site for DNMT1 [118].

### 2.3. A Conciliated Model of How the UHRF1/DNMT1 Tandem Works

In light of all the studies, the following question remains: what is the correct model for the UHRF1-dependent CpG targeting by DNMT1? The majority of these studies were performed on purified isolated proteins, thus questioning the relevance of the *in vivo* occurring. However, if both models are right, we have to find an explanation to render them compatible.

We, therefore, propose a more complete model in which UHRF1 does not recruit DNMT1, but in which they are still together in the same large macro-molecular complex; thus, DNMT1 is in permanent presence, in close vicinity of UHRF1, as long as this complex progresses at the DNA replication fork, although a direct transfer of a CpG site from the SRA to DNMT1 appears not applicable [117]. When the SRA domain meets a hemi-methylated CpG dinucleotide, UHRF1 adopts an open conformation allowing ubiquitination of histone H3 and/or auto-ubiquitination via the RING, facilitated by the cooperation of the UBL and E2 Ube2D (ubiquitin-conjugating enzyme E2D). The ubiquitination of histone H3 and/or UHRF1 auto-ubiquitination represent an anchorage signal for the RFTS domain of DNMT1, liberating the catalytic activity of DNMT1. Meanwhile the UHRF1/TTD tandem binds to the methylated histone H3 at lysine 9. This first step is preparing DNMT1 to enter in action, i.e., to methylate the new DNA strand; however, this is not sufficient to localize it. We further propose that UHRF1, when flipping the mC through the SRA domain may undergo an allosteric change that allows creating contact zones with DNMT1 inside the large complex they belong to. This interaction facilitates accession of the catalytic center of DNMT1 to its substrate, i.e., to hemi-methylated DNA, along with the release to mC by the SRA domain, considering that steric hindrance forbids that both mCs on each DNA strand can be flipped at the same time by UHRF1 and DNMT1 [119]. We do not exclude that, when UHRF1 releases the mC, it adopts a conformational change which promotes the seeking of other hemi-methylated CpG sites, leaving behind DNMT1 to methylate the newly synthesized DNA strand. We do not exclude either that the RFTS domain, chronologically, may firstly interact with ubiquitinated histone H3 and, secondly, with the SRA domain in order to remove UHRF1 from the CpG dinucleotide, considering that DNMT1 cannot methylate the opposite DNA strand as long as UHRF1 is present on the same targeted hemi-methylated CpG.

Our model supports a repetitive dialogue between DNMT1 and UHRF1 during the synthesis (S) phase at CpG sites, and is in opposition to a model in which DNMT1 leaves the chromatin as soon as it catalyzes one enzymatic reaction, i.e., a hydrogen atom replaced by a methyl group on a cytosine. This model is particularly likely when CpG islands (repeated methylated CpG) are methylated. An interesting question arises from this model: what happens between two methylated CpG dinucleotides or between two methylated CpG islands? In our opinion, it is unlikely that DNMT1 dissociates from UHRF1 or the UHRF1 complex after each hemi-methylated CpG dinucleotide. Otherwise, it would take too much time for UHRF1 to wait for recruiting (i.e., to find) a molecule of DNMT1; furthermore, what would be the signal telling DNMT1 to join the UHRF1 complex? All that would hinder the epigenetic code replication machinery, in which UHRF1 plays the role of conductor, to follow the progression of the DNA replication fork as fast as required. In contrast, a more plausible behavior of DNMT1 and UHRF1 would be that they are in close contact in the complex while moving along the DNA, and when UHRF1, through the SRA domain, flips out the methylated cytosine, it probably communicates with DNMT1 to prepare it for recognition of the unmethylated cytosine on the opposite DNA strand. Due to allosteric constraints, UHRF1 needs to further progress to allow DNMT1 to exert its catalytic activity. Accordingly, it was proposed that the removal of the RFTS from the enzymatic pocket of DNMT1, by UHRF1, is involved in its progressive methylation property [114]. When the DNMT1/UHRF1 complex progresses along the DNA, without meeting hemi-methylated DNA, the SRA domain may not interact with the RFTS domain of DNMT1 and, in this way, DNMT1 has no enzymatic activity, avoiding undesired or unfaithful DNA methylation. However, UHRF1 may still interact with DNMT1, but through other domains previously identified [24], and this state would be a kind of standby. An interesting hypothesis would be that this standby also occurs during development, during which a faithful inheritance of DNA methylation patterns would be a brake for cell differentiation or would be an undesired event [67].

## 3. Concluding Remarks

In conclusion, we propose that both models are correct and compatible, but the events they are based on may be separated in time, with model A most probably happening first and model B happening just after. The following chronological model of the dialogue between UHRF1 and DNMT1 to ensure faithful inheritance of DNA methylation patterns is, therefore, proposed: hemi-methylated DNA–SRA interaction; binding of the TTD to H3K9me3; spacer facilitation of ubiquitination by the RING domain of a dual combination of lysines 14,18, and 23 of histone H3, and probably also UHRF1 auto-ubiquitination; recruitment (or preferred term interaction) of DNMT1 with mono-ubiquitinated histone H3 and/or ubiquitinated UHRF1; interaction between the SRA domain and/or UBL domain with DNMT1 (via the RFTS domain) and alleviation of the auto-inhibition of DNMT1; and, finally, methylation of the newly synthetized DNA by DNMT1 (Figure 2a,b). This model, however, is based on experimental data obtained with purified proteins, which is a situation that never occurs in vivo as evidenced by numerous studies, reporting that UHRF1 belongs to a macro-molecular complex containing a high number of partners [25,105,120]. This large macro-molecular complex is closely located to the replication fork as it associates with PCNA [18,19,83,121]. We suggest that the UHRF1/DNMT1 tandem slides along the newly synthesized DNA, immediately following the DNA replication machinery in a perpetual complex interacting mode, governed by the status of hemi-methylation of the DNA. The two models of the inheritance of DNA methylation patterns are both likely to ensure faithful DNA methylation duplication. We do not exclude that the two models can act as a kind of double check or double lock to secure fidelity of transmission of methylated profiles. Technological challenges are present that must be overcome to unravel the complexity of the dialogue (direct and/or indirect communication) between UHRF1 and DNMT1. Investigations of in situ domain–domain interactions between the two proteins will help understand when and how each model occurs. This is the price to pay to progress in the understanding of the transmission of this important epigenetic mark.
